# Transcranial direct current stimulation combined with speech therapy in Fragile X syndrome patients: a pilot study

**DOI:** 10.3389/fneur.2023.1268165

**Published:** 2023-12-05

**Authors:** Chiara Picciuca, Martina Assogna, Romina Esposito, Alessia D’Acunto, Matteo Ferraresi, Silvia Picazio, Ilaria Borghi, Alex Martino Cinnera, Sonia Bonnì, Pietro Chiurazzi, Giacomo Koch

**Affiliations:** ^1^Department of Behavioral and Clinical Neurology, Santa Lucia Foundation IRCCS, Rome, Italy; ^2^Department of Systems Medicine, University of Rome Tor Vergata, Rome, Italy; ^3^Department of Neuroscience and Rehabilitation, University of Ferrara, Ferrara, Italy; ^4^Department of Movement, Human and Health Sciences, University of Rome "Foro Italico", Rome, Italy; ^5^Sezione di Medicina genomica, Dipartimento di Scienze della Vita e Sanità Pubblica, Università Cattolica del Sacro Cuore, Rome, Italy; ^6^UOC Genetica Medica, Fondazione Policlinico Universitario "A. Gemelli" IRCCS, Rome, Italy

**Keywords:** tDCS, FXS, speech therapy, prefrontal cortex, TMS-EEG

## Abstract

**Background:**

Fragile X syndrome (FXS) is the leading cause of genetic intellectual disability. Among the neurobehavioral dysfunctions in FXS individuals, language development and literacy are compromised. Recent evidence hypothesized that the disruption of excitatory glutamatergic and GABAergic inhibitory neurotransmission balance might be responsible for impairment in cognitive function. In this study, we evaluated for the first time, the safety, tolerability, and efficacy of anodal prefrontal transcranial direct current stimulation (tDCS) combined with standard speech therapy to enhance language function in FXS patients.

**Methods:**

In total, 16 adult FXS patients were enrolled. Participants underwent 45 min of anodic tDCS combined with speech therapy for 5 weeks (3 times per week). Language function was evaluated using the Test for Reception of Grammar–Version 2 (TROG-2) and subtests of the Italian Language Examination (Esame del Linguaggio – II, EDL-II). Right and left dorsolateral prefrontal cortex transcranial magnetic stimulation and concurrent electroencephalography (TMS-EEG) recordings were collected at baseline and after the treatment to evaluate cortical reactivity and connectivity changes.

**Results:**

After 5 weeks of combined therapy, we observed a significant improvement in the writing (7.5%), reading (20.3%), repetition (13.3%), and TROG-2 (10.2%) tests. Parallelly with clinical change, TMS-EEG results showed a significant difference in TMS-evoked potential amplitude over the left frontal cortex after treatment (−0.73 ± 0.87 μV) compared to baseline (0.18 ± 0.84 μV).

**Conclusion:**

Our study provides novel evidence that left anodal prefrontal tDCS combined with standard speech therapy could be effective in enhancing language function in FXS patients, mainly by inducing a rebalance of the dysfunctional prefrontal cortical excitability.

## Introduction

Fragile X syndrome (FXS) causes inherited mild-to-severe intellectual disability and is the most frequent monogenic cause of autism spectrum disorder (ASD) worldwide (1, 2). FXS arises from a loss of function mutation in the X-linked *FMR1* gene due to its transcriptional silencing resulting from hypermethylation of an abnormally expanded CGG trinucleotide repeat in the 5′ untranslated region (3). The transcriptional silencing of *FMR1* results in the absence or reduction of Fragile X messenger ribonucleoprotein (FMRP), which is an RNA-binding protein involved in synapse maturation and neural circuit function (4). Accordingly, a disruption in functional integration within brain networks occurs and accounts for neurobehavioral dysfunctions in FXS individuals, which range from developmental delay with general cognitive impairment and severe disability to milder cases with impulsivity, increased response to sensitive stimuli, social anxiety and phobias, hyperactivity, attention deficit, and autism spectrum disorder (5). Language development and literacy are compromised in FXS individuals, with language delays that have been described in the areas of overall communication abilities and the specific domain of expressive, receptive, and pragmatic language and speech intelligibility (6, 7). Common language deficits observed in FXS individuals include delayed language development, articulation deficits, and language processing difficulties, with a consequent impact on social communication skills. The pathophysiological alterations that underlie language deficits in Fragile X syndrome are not fully understood, but a previous study suggests a key role of functional connectivity disruption between the frontal and temporal cortex and exaggerated frontal gamma power before speech onset (8). Accordingly, in the last few years, neurotransmission and synaptic deficits have been shown in the Fmr1 knockout (KO) mouse models, which show common phenotypes with FXS patients, opening new potential targets for therapeutic interventions (4, 9). Indeed, a key function of FMRP is to inhibit protein translation at the synapse, with a consequent upregulation of metabotropic glutamate receptors (mGluRs) 1 and 5 and exaggerated hyperexcitability and long-term depression (LTD) mechanisms (10). Defects in the inhibitory GABAergic system have also been identified in the amygdala, cerebral cortex, and cerebellum in *Fmr1* knockout mice models, and they are associated with symptoms such as seizures, anxiety, and attention processing deficit (11), which characterize FXS (12–15). Current pharmacological treatments available for FXS are limited to the use of antiglutamatergic drugs, mood stabilizers, and serotonin reuptake inhibitors to control anxiety and depression. However, no effective treatment has been identified to treat cognitive and autistic behavior, with interventions mainly focused on symptom management. Non-invasive brain stimulation techniques have been widely used to investigate the neurophysiological basis of neurological conditions and to treat several neuropsychiatric symptoms (16–18). Anodal prefrontal transcranial direct current stimulation (tDCS) applied over language areas including Broca’s area has been used to improve articulation and speech output in autism spectrum disorder patients (19) and individuals with chronic aphasia (20–23). Based on these premises, the current study aimed to evaluate the safety, tolerability, and efficacy of prefrontal tDCS combined with standard speech therapy sessions to enhance linguistic function in FXS patients. To detect possible *in vivo* neurophysiological changes in cortical excitability and cortical oscillations underlying cognitive effects, we collected transcranial magnetic stimulation combined with electroencephalography (TMS-EEG) recordings over the prefrontal cortex.

## Methods

### Subjects

This was an open-label, prospective, longitudinal pilot study. Sixteen adults (26.9 ± 10.11 years, 1 F) with Fragile X syndrome were enrolled in this study (complete demographic and baseline clinical data are available in Table 1). The study was conducted from May 2021 to December 2021 at the Santa Lucia Foundation Hospital. We enrolled all patients with a molecular diagnosis of FXS that meet the following inclusion criteria: (*i*) FXS diagnosis with full mutation of the FMR1 gene; (*ii*) age between ≥18 and ≤ 50 years; and (*iii*) stable pharmacological therapy at least 30 days before enrollment. We excluded each patient with contraindications for transcranial electrical stimulation and/or concomitant diseases: (*i*) history of seizures; (*ii*) intracranial metal implants; (*iii*) cardiac pacemaker; (*iv*) pregnancy status; and (*v*) concomitant diseases. Written informed consent was obtained from each participant and their legal guardian. The study was performed according to the Declaration of Helsinki, and it was approved by the Ethics Committee of Santa Lucia Foundation Hospital (Prot. CE/PROG.933).

**Table 1:** Demographical characteristics and baseline language scores of Fragile X patients.

Subject	Sex	Age	Copy	TROG-2	Reading	Writing	Repetition
1	M	24	9	59	87	83	98
2	M	24	0	15	0	0	15
3	M	22	10	38	53	36	93
4	M	27	10	59	85	75	99
5	M	19	10	40	16	19	93
6	M	23	0	5	0	0	42
7	F	19	10	61	96	93	98
8	M	43	9	46	82	73	100
9	M	47	10	64	95	93	100
10	M	21	10	46	91	90	100
11	M	36	10	27	52	18	96
12	M	46	4	28	41	24	99
13	M	21	10	74	98	99	100
14	M	18	8	33	65	63	96
15	M	23	10	40	0	0	95
16	M	18	4	41	0	1	79

M, male; F, female; TROG-2, test for reception of grammar – version 2. Copy: total score = 10; TROG-2: total score = 80; Reading: total score = 100; Writing: total score = 100; Repetition: total score = 100.

### Experimental design

**Figure 1:**
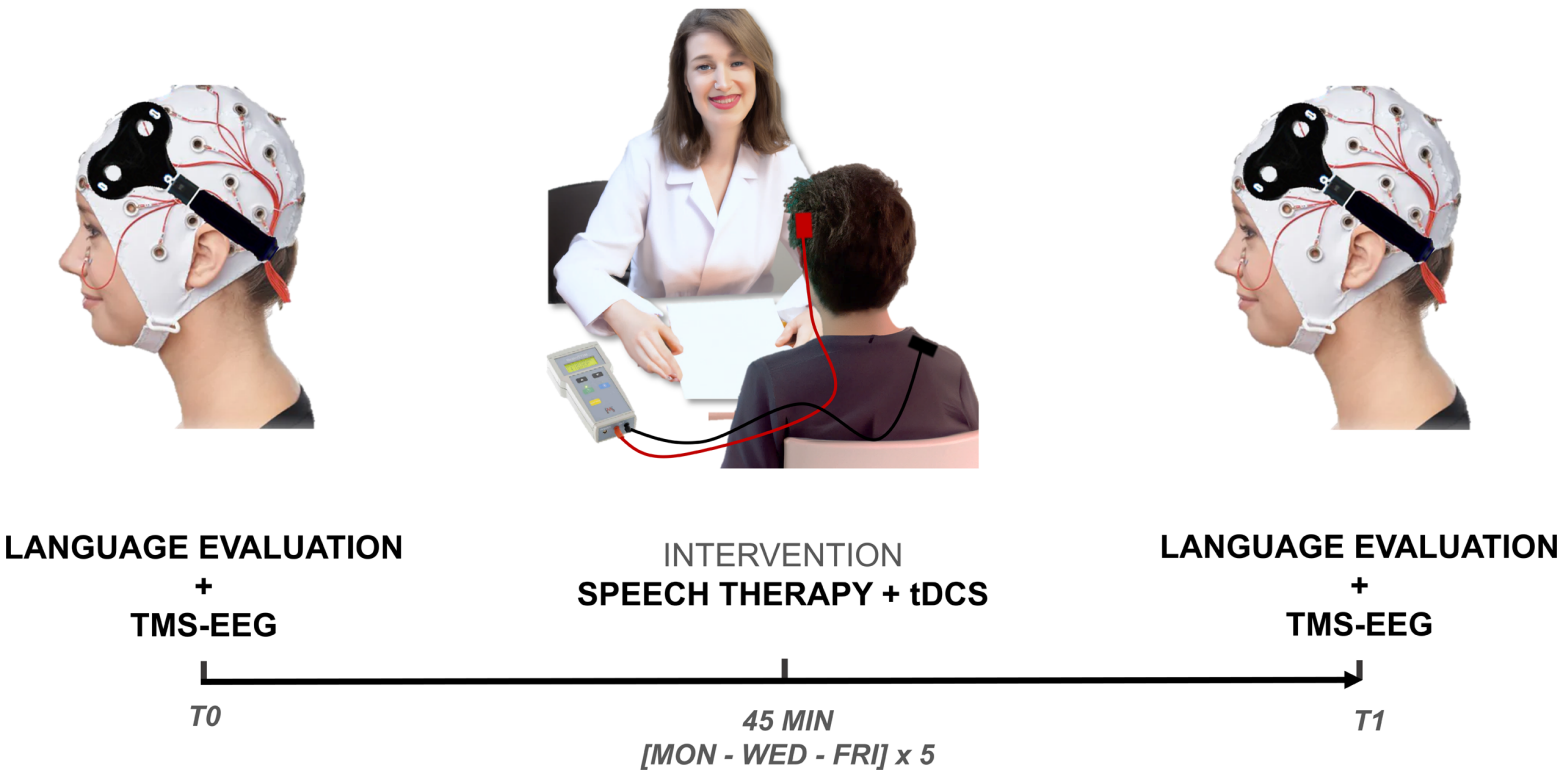
Experimental design. Patients were evaluated with a specific neuropsychological battery and TMS-EEG over l and r-DLPFC before (T0) and after (T1) the treatment protocol. During the treatment protocol, patients were exposed to a period of 45 min stimulation with anodic tDCS combined with speech therapy for 5 weeks, with a frequency of three times per week, for a total of 15 treatments. TMS-EEG, transcranial magnetic stimulation combined with electroencephalography; tDCS, transcranial direct current stimulation.

Participants were undergoing 45 min of stimulation with anodal tDCS combined with speech therapy for 5 weeks, with a frequency of three times per week, for a total of 15 treatments. Patients underwent therapy on Monday, Wednesday, and Friday. The speech therapy lasted 1 h per session, and it was conducted concurrently with tDCS for the first 45 min, after which speech therapy was administered exclusively for the last 15 min (all sessions were administered by C.P.). A formed administer (I.B.) assessed linguistic changes in patients with a specific neuropsychological battery before (T0) and right after (T1) the treatment protocol. TMS-EEG recordings on the left and right dorsolateral prefrontal cortex (DLPFC) were collected to evaluate potential changes in prefrontal cortical excitability and TMS-evoked oscillatory activity. A schematic representation of the experimental design is depicted in Figure 1.

### Neuropsychological evaluation

All patients underwent a neuropsychological evaluation for language disorders including the following tests.

#### Word, non-word, and phrase reading test

Patients were required to read aloud 20 words divided into high-and low-frequency words and selected for length (4/6 phonemes and more than 6 phonemes); 20 non-words (monosyllabic, disyllabic, and trisyllabic); and 10 phrases. Reading errors were scored by an experimenter. The total score was 40 points for words; 40 points for non-words; and 20 points for phrases (24). The total score ranged from 0 to 100 points; a higher score represents an improvement in the reading skills.

#### Word, non-word, and phrase writing test

Patients were required to write under dictation 20 words divided into high-and low-frequency words and selected for length (4/6 graphemes and more than 6 graphemes); 20 non-words (monosyllabic, disyllabic, and trisyllabic); and 10 phrases. Writing errors were scored by an experimenter and analyzed as the errors of “word, non-word, and phrases reading test.” The total score was 40 points for words; 40 points for non-words; and 20 points for phrases (24). The total score ranged from 0 to 100 points; a higher score represents an improvement in the writing skills.

#### Word, non-word, and phrase repetition test

Patients were required to repeat aloud 20 words divided into high-and low-frequency words and selected for length (4/6 phonemes and more than 6 phonemes); 20 non-words (monosyllabic, disyllabic, and trisyllabic); and 10 phrases. Repetition errors were scored by an experimenter and analyzed as the errors of “word, non-word, and phrases reading test.” The total score was 40 points for words; 40 points for non-words; and 20 points for phrases (24). The total score ranged from 0 to 100 points; a higher score represents an improvement in the repetition skills.

#### Word copy test

Patients were required to copy five words. In total, 2 points were assigned for a correct copy; 1 point was assigned for a letter error; and 0 points for other cases (24). The total score ranged from 0 to 10 points; a higher score represents an improvement in the copy skills.

#### Test for reception of grammar – version 2

TROG-2 (25) is a multiple-choice sentence picture-matching task where the participants listen to a spoken sentence and must select one of four pictures to match what is heard. Items are organized into 20 blocks of 4 items each, with the grammatical complexity of the blocks increasing as the test progresses. The total score ranged from 0 to 80 points; a higher score represents an improvement in the reception of grammar ability.

### TMS-EEG recordings

Patients underwent TMS-EEG recordings before (T0) and right after (T1) during the treatment period to evaluate cortical reactivity and oscillatory activity. For each session, 80 TMS single-pulses were applied at a random ISI of 2–4 s, with a variation of 20%, over right and left DLPFC, targeted by using the 10–20 system by placing the TMS coil over F3 for the right DLPFC and F4 electrode for left DLPFC, respectively. Stimulation intensity was set at 110% of resting motor threshold (RMT), defined as the lowest intensity-evoking MEPs of > 50 μV in at least five out of ten trials in the relaxed first dorsal interosseous (FDI) muscle of the hand contralateral to the stimulation (26). The order of stimulation of the two areas was randomized across patients and time points. We used a TMS-compatible DC amplifier (BrainAmp, Brain Products GmbH, Munich, Germany) to record continuous EEG activity from the scalp. The EEG was recorded from 64 sites positioned according to the 10–20 International System, using TMS-compatible Ag/AgCl pellet electrodes mounted on an elastic cap. EEG signals were digitized at a sampling rate of 5 kHz. TMS-EEG data were preprocessed offline (27). Data were segmented into epochs starting 1 s before the TMS pulse and ending 1 s after the pulse. A cubic interpolation was applied from 1 ms before to 10 ms after the TMS pulse to remove TMS-induced artifacts. Next, the signal was downsampled from 5,000 Hz to 1,000 Hz. Afterward, a high-pass filter at 1 Hz was applied to the continuous data. Subsequently, the low-pass filter at 90 Hz and a notch filter at 50 Hz was applied to the data. Next, all the epochs were visually inspected, and the EEG epochs containing artifacts or noisy signals were rejected. Physiological and TMS-related artifact components were detected using Fast-ICA and removed based on their scalp distribution, frequency, timing, and amplitude. All data were re-referenced to the average of all scalp channels, and residual epochs containing artifacts were removed during a second visual inspection. Finally, the signal was imported into Fieldtrip (28). Baseline correction was applied using the pre-TMS interval from-100 ms to-1 ms.

### Speech therapy: phonological reading and writing remediation program

A remediation program (29) was applied to the patients. This program is based on grapheme-phoneme conversion. Treatment was divided into three distinct steps, with 15 cumulative sessions, three times a week, with an average duration of 1 h each.

*Step 1: Phonological (5 Sessions).* This step involved auditory discrimination, the addition and subtraction of phonemes and syllables, and syllabic and phonemic manipulation.
*Step 2: Phonological and Reading (5 Sessions).* This step involved auditory discrimination, the addition and subtraction of phonemes and syllables, syllabic and phonemic manipulation, letter and phoneme identification, rapid naming of letters, visual discrimination, and reading of stories for oral comprehension.
*Step 3: Phonology, Reading and Writing (5 Sessions).* This step involved auditory discrimination; the addition and subtraction of phonemes and syllables; syllabic and phonemic manipulation; identification of letters and phonemes; rapid naming of letters; visual discrimination; oral reading of stories for oral comprehension; dictation of syllables, real words, and non-words; dictation of sentences; and dictation of texts and recount writing stories.

### Transcranial direct current stimulation

tDCS was generated using a BrainStim stimulator by E.M.S. s.r.l. (Bologna, Italy) and delivered via a pair of identical, square, scalp electrodes (5 × 5 cm^2^) made of conductive rubber and covered with saline-soaked synthetic sponges. The anodal electrode was positioned over the left prefrontal cortex (L-DLPFC) according to the 10–20 EEG on the sites corresponding to F3. The cathodal electrode was placed over the right deltoid muscle (30, 31). At the beginning of the active tDCS, the current was increased slowly during the first 30 s to 2 mA at the stimulation threshold (ramp-up) and, at the end of the stimulation, the current was decreased slowly to 0 mA during the last 30 s (ramp-down), with a 0.08 mA/cm^2^ current density (32). Between the ramp-up and ramp-down constant, direct current (2 mA) was delivered for 45 min.

### Statistical analysis

Data were analyzed using SPSS version 22 (SPSS Inc., Chicago, IL, United States). The Shapiro–Wilk test was used to assess the normal distribution of neuropsychological data. The level of significance was set at α = 0.05. To assess the effects of tDCS combined with speech therapy effects on patients’ neuropsychological evaluation, we used the Wilcoxon non-parametric test to compare the performance before the treatment (“pre-treatment”) and right after it (“post-treatment”), separately for each test. The relationship between variables was computed by Spearman’s rho coefficient. We aimed to evaluate whether the TEPs recorded before and after the treatment differed in amplitude over space and time for both stimulation conditions. Then, we assessed the differences in the time-frequency domain. For these analyses, we conducted non-parametric cluster-based permutation tests to correct for multiple comparisons as implemented by the ft_timelockstatistics and ft_freqstatistics functions in Fieldtrip (28) with MATLAB (MathWorks Inc., Natick, MA, 2021b). The neurophysiological comparisons involved all the electrodes for each stimulation condition for the time window of interest from 11 to 60 ms after the TMS pulse. The definition of the time window was based on preventing artifacts, i.e., auditory evoked potentials at 100 ms (33–35). Based on the initial one-sample *t*-tests, all *t*-values above a threshold, corresponding to an uncorrected *p*-value of 0.05, were grouped into clusters based on adjacent significant time points and electrodes, considered separately, for a sample with positive and negative *t*-values (two-tailed test). Subsequently, this procedure was repeated across 2,500 permutations by calculating Monte Carlo estimates of the significance probabilities (*p* < 0.05). To evaluate changes in the oscillatory domain, we performed a time-frequency decomposition based on the Morlet wavelet (number of cycles =7, 1 Hz steps from 4 to 80 Hz). Then, we computed non-parametric cluster-based permutation tests to correct for multiple comparisons. The neurophysiological comparisons involved all the electrodes for each stimulation condition for two different time windows of interest from 0 to 300 ms and from 0 to 100 ms after the TMS pulse, respectively. The same analysis has been applied to the stimulated electrodes, F3 and F4, for the left and right prefrontal stimulation conditions, respectively. Based on the initial one-sample *t*-tests, all *t*-values above a threshold, corresponding to an uncorrected *p*-value of 0.05, were grouped into clusters based on adjacent significant time points and electrodes, considered separately, for a sample with positive and negative *t*-values (two-tailed test).

## Results

All procedures are well tolerated with complete adherence to the treatment when each patient completes the 15 sessions. Regarding assessment, one patient was not compliant with the TMS-EEG evaluation. tDCS procedures were well tolerated, and no adverse events (AEs) were reported. The tDCS is a safe technique to perform neuromodulation, in the literature are not reported any reports of serious AEs or irreversible injury (36). Slight side effects were reported in other studies in the form of burn-like lesions and contact dermatitis. Mania or hypomania was also reported in unipolar and bipolar depression patients without a clear causal relationship between tDCS treatments. Finally, an isolated case of seizure was reported in the literature but the involvement of tDCS was not confirmed (37).

After 5 weeks of treatment (tDCS coupled with speech therapy), we observed a significant improvement in the word, non-word, and phrase writing test scores in the post-treatment evaluation with respect to the baseline evaluation (T0: 47.94 ± 38.99; T1: 58.13 ± 40.28; Z = −2.937; *p* = 0.004; *r* = 0.519). We also found a significant improvement in the word, non-word, and phrase reading test scores (T0: 53.81 ± 39.15; T1: 67.12 ± 38.55; Z = −3.297; *p* = 0.001; *r* = 0.58) and in the word, non-word, and phrase repetition test scores (T0: 87.69 ± 24.18; T1: 91.63 ± 19.77; Z = −3.069; *p* = 0.002; *r* = 0.54). Moreover, we observed a statistically significant amelioration in the TROG-2 test score after the treatment (T0: 42.25 ± 18.44; T1: 58.5 ± 17.56; Z = −3.53; *p* < 0.001; *r* = 0.624). Finally, we found a trend toward increasing the copy test core post-treatment compared to the pre-treatment evaluation (T0: 7.75 ± 3.62; T1: 8.5 ± 3.46; *p* = 0.057), and the complete results and effectiveness improvement are reported in Table 2.

**Table 2:** Cognitive effects of the combined tDCS and speech therapy on language function in FXS patients.

	Pre-treatment	Post-treatment	Effectiveness
Writing (M ± SD)	47.94 ± 38.99	58.13 ± 40.28^**^	7.5%
Reading (M ± SD)	53.81 ± 39.15	67.12 ± 38.55^***^	20.3%
Repetition (M ± SD)	87.69 ± 24.18	91.63 ± 19.77^**^	13.3%
TROG-2 (M ± SD)	42.25 ± 18.44	58.5 ± 17.56^***^	10.2%
Copy (M ± SD)	7.75 ± 3.62	8.5 ± 3.46	3.9%

tDCS, transcranial direct current stimulation; TROG-2, test for reception of grammar – version 2; FXS, Fragile X syndrome; M, mean; SD, standard deviation. The effectiveness was computed as follows: $\frac{T1-T0}{\mathrm{MAXscore}}*100$ ; where “*T*1−*T*0” is the difference between the post treatment time-point and the baseline, and *MAX_score_* is the maximum score for the evaluated outcome measure (38). The statistical significance level of the Wilcoxon non-parametric test has been highlighted as follows: ^*^ *p* ≤ 0.05; ^**^ *p* ≤ 0.01; ^***^ *p* ≤ 0.001.

**Figure 2:**
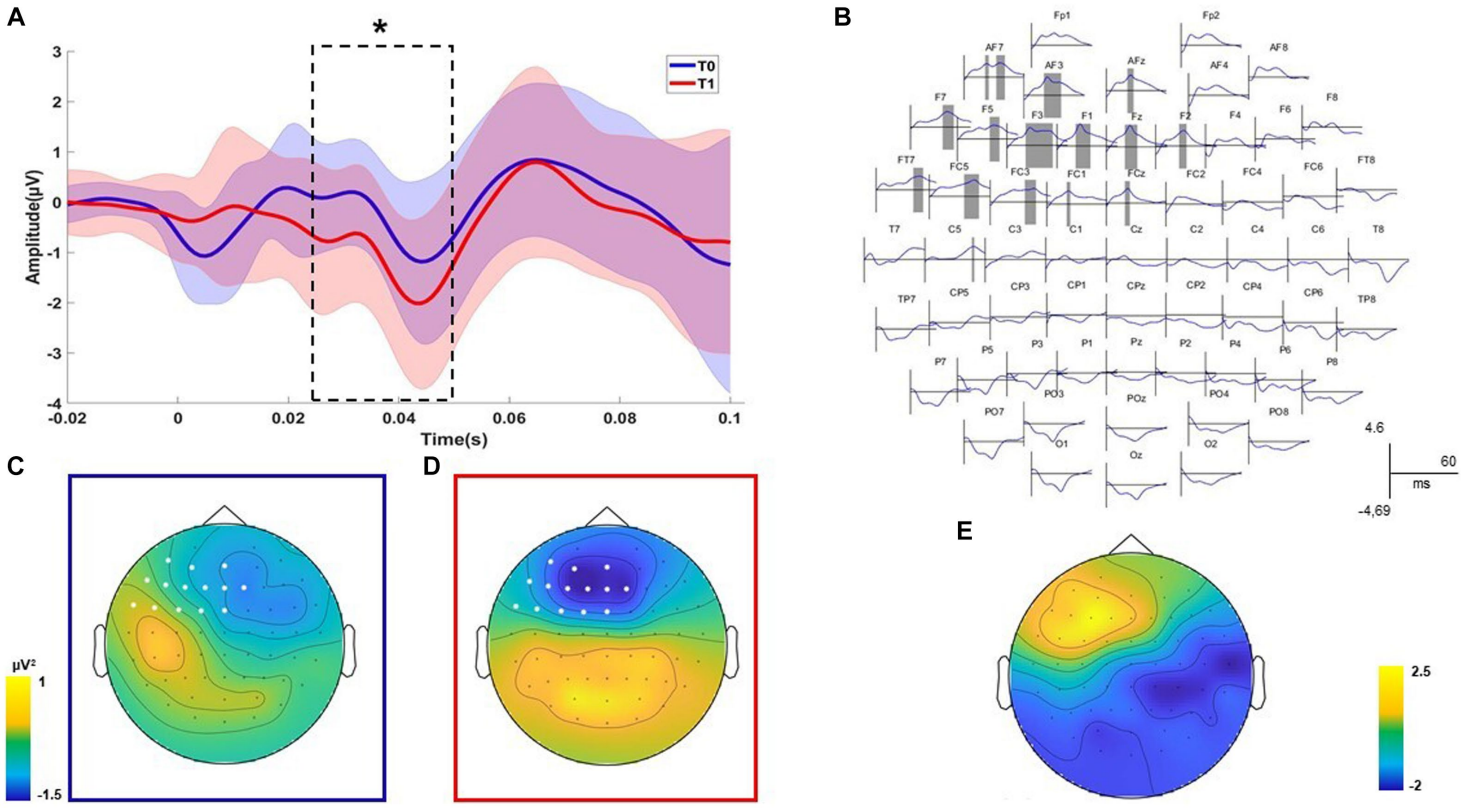
Neurophysiological results. Significant positive cluster of comparisons before and after the treatment for the DLPFC stimulation condition is shown. The plot of the averaged electrodes, with the respective standard deviation, which are part of the cluster is displayed **(A)**. Dashed lines indicate the temporal extension of each significant cluster **(B)**. The topographies on the left indicate the voltage of the signal over all electrodes for the significant time window and indicate the electrodes that are part of the cluster **(C,D)**. The topographies on the right indicate the scalp distribution of the cluster *R* values **(E)**. Statistical significance level of the Wilcoxon non-parametric test has been highlighted as follows: ^*^ *p* ≤ 0.05; ^**^ *p* ≤ 0.01; and ^***^ *p* ≤ 0.001.

TMS-EEG analysis revealed a significant positive cluster (*p* = 0.047) relating to the differences before and after the treatment recorded with the right DLPFC stimulation condition (Figure 2). The positive cluster included the signal from left frontal electrodes (AF7, AF3, AFz, F7, F5, F3, F1, Fz, F2, FT7, FC5, FC3, FC1, FCz, and C5) in the time interval of 26 to 51 ms (Figures 2A,B). Considering that the signal post-treatment of this cluster was negative on average, as shown in the topography in Figures 2C,D, this result indicated that the TEP amplitude in left frontal sites decreased after the treatment concerning the baseline. The statistical comparison performed on all electrodes in the time window from 7 to 60 ms after the TMS pulse did not reveal any spatiotemporal differences for the left DLPFC stimulation condition (all *p* > 0.05). For the time-frequency analysis, any cluster results were not significant for both the stimulation conditions (all *p* > 0.05).

Post-treatment neuropsychological measures (reading, writing, repetition, word copy, and TROG-2) have been correlated with the mean values of significant clusters emerging from neurophysiological comparisons. A negative correlation between the total scores of the reading test (EDL-II) and the F2 component was found (*R* = − 0.573; *p* = 0.025). A negative correlation between the total scores of the writing test (EDL-II) and the F2 component was found (*R* = − 0.654; *p* = 0.008). Finally, a negative correlation between the total scores of the TROG-2 Test and the AFz component was found (*R* = − 0.549; *p* = 0.034). Overall, correlational analysis results showed that patients with better linguistic performances after treatment had reduced frontal excitability.

## Discussion

In this study, we evaluated the efficacy of left prefrontal tDCS combined with standard speech therapy to enhance language function in FXS patients. We found that anodal left prefrontal tDCS combined with speech therapy was able to induce a significant amelioration in writing, reading, repetition, and TROG-2 comprehension test scores in our population. Our neurophysiological data suggest that this improvement is accompanied by a parallel decrease after treatment in left prefrontal excitability. Moreover, correlational analysis showed that patients with lower left prefrontal cortical excitability displayed better language function enhancement after treatment. These results indicate that the modulation of the prefrontal cortex induced by the anodal tDCS intervention could be effective in individuals with FXS, and further highlight the alteration between excitatory and inhibitory circuits balance as a target for intervention in individuals with FXS. Our results are in line with previous studies suggesting that the disruption of excitatory glutamatergic and GABAergic inhibitory neurotransmission balance might be responsible for impairment in cognitive function, selective attention deficits, and many of the other behavioral and developmental aspects of FXS, as well as for language deficits (8, 14, 15). Accordingly, a recent study has shown that individuals with FXS often exhibit atypical neurophysiological patterns, including increased activation in the frontal region of the brain during language processing and a negative correlation between the degree of frontal activation and language performance in individuals with FXS (8). This suggests that as frontal activation increases, language abilities tend to decline or remain impaired. Conversely, when frontal activation decreases, there may be an improvement in language skills, as already shown in autism spectrum disorder (39). With this regard, tDCS has been previously shown to modulate functional connectivity between DLPFC and other regions of the brain (40), and this additional mechanism could suggest another possible neurophysiological explanation for our results. In line with this observation, our findings might indicate that excessive left prefrontal activation or over-engagement in the prefrontal region during language processing could potentially hinder language performance. Reduced frontal activity may allow for more efficient neural processing or allocation of cognitive resources to other language-related regions, leading to enhanced language skills. Beneficial cognitive effects induced by tDCS in language function might depend also on improved learning or working-memory modulation, as previously described (41). Recent studies have demonstrated that anodal tDCS over DLPFC was able to enhance naming and speed verbal reaction time performances, thus leading to hypothesize its role in a specific network dedicated to lexical retrieval/selection processing in naming (42). Moreover, the combination of anodal tDCS in Broca’s area with standard speech therapy has been tested in the aphasic patient population with beneficial effects in terms of articulatory defects (21), naming accuracy (43), and verbal fluency (44). In addition, tDCS may also exert non-neuronal effects in FXS, possibly by enhancing the expression of brain-derived neurotrophic factor (BDNF). The BDNF is a neurotrophic factor that supports neurogenesis and survival of neurons by modulating the inflammatory response acting on microglia and reducing the interference with blood–brain barrier disruption (45). Accordingly, microglia play a critical role in the development and maintenance of synapses through bidirectional communication, and its alteration was demonstrated to be a potential contributor to the pathophysiology of FXS (46). Overall, to the best of our knowledge, this was the first study showing high feasibility, tolerability, and safety of tDCS in a cohort of the FXS population. All the participants completed the treatment session successfully, and no significant discomfort was reported at the electrode sites and no side effects were observed, such as seizures, acute mood changes, and irritability. Eleven patients (out of sixteen) reported a persistent tingling sensation under electrodes which did not affect the tDCS and speech therapy session. Although most children with FXS start speech-language intervention early in their lives, there are no current studies that have specifically evaluated language interventions for individuals with FXS. Recent evidence suggests that speech therapy programs that have been designed broadly for individuals with language learning difficulties, including individuals with intellectual and developmental disabilities, may be appropriate for individuals with FXS, even for older children (47, 48). In this context, our study provides novel evidence that combining speech therapy intervention with neuromodulation could be effective in FXS individuals; however, more clinical studies evaluating standard therapy approaches alone for this population are needed. Surprisingly, in our study, we found an enhancement of language function after the combined treatment in an adult sample of FXS patients with a great heterogeneity in language skills. The two non-verbal adults showed progress in auditory discrimination in phonemic and syllabic production, and this is an important result since this trial permits them to approach verbal communication. Twelve participants showed improvement in reading and writing ability as well as in receptive language, and caregivers believe that after treatment, individuals with FXS are more interested in communicating through reading and writing, e.g., they write spontaneously the means of public transport or the name of product and price in the supermarket. Finally, the two high-functioning participants showed improvements in oral reading of stories for oral comprehension, dictation of syllables, real words and non-words, dictation of sentences, dictation of texts, and recounting writing stories, and they were more skillful in studying university books. Our study is limited by the absence of a sham-controlled group and by the small sample of patients. However, we used neurophysiological measures to detect possible cortical changes underpinning cognitive functioning, and our findings are in line with recent literature, which shows that lower left prefrontal cortical excitability displayed better language function enhancement after treatment. Moreover, our conclusion is limited by the lack of assessment of the effects of either prefrontal tDCS or speech therapy separately and the lack of long-term follow-up. Further studies with a randomized, double-blind sham-controlled trial design and a longer follow-up are needed to optimize non-invasive brain stimulation combined with speech therapy in FXS patients.

## Conclusion

In conclusion, our study provides evidence that left prefrontal tDCS combined with standard speech therapy might be effective in enhancing language function in FXS patients, mainly through the reduction of frontal cortical hyperexcitability. Considering the impact of language disturbances in FXS individuals and their families and the lack of pharmacological treatment or specific speech intervention, our results suggest that neuromodulation strategies targeting excitation/inhibition neurotransmission imbalance and connectivity disruption could represent a potential new therapeutic tool in FXS.

## Data availability statement

The original contributions presented in the study are included in the article/supplementary material, further inquiries can be directed to the corresponding author.

## Ethics statement

The studies involving humans were approved by Santa Lucia Foundation Ethics Committee. The studies were conducted in accordance with the local legislation and institutional requirements. Written informed consent for participation in this study was provided by the participants’ legal guardians/next of kin. Written informed consent was obtained from the individual(s) for the publication of any potentially identifiable images or data included in this article.

## Author contributions

CP: Data curation, Investigation, Project administration, Writing – original draft. MA: Writing – original draft, Formal analysis, Visualization. RE: Formal analysis, Writing – review & editing. AD: Writing – review & editing, Data curation. MF: Data curation, Writing – review & editing. SP: Writing – review & editing, Conceptualization, Formal analysis. IB: Writing – review & editing, Visualization. AMC: Visualization, Writing – review & editing. SB: Visualization, Writing – review & editing. PC: Visualization, Writing – review & editing. GK: Writing – review & editing, Conceptualization, Methodology, Supervision.
